# New Food Frequency Questionnaire to Estimate Vitamin K Intake in a Mediterranean Population

**DOI:** 10.3390/nu15133012

**Published:** 2023-07-01

**Authors:** Ezequiel Pinto, Carla Viegas, Paula Ventura Martins, Tânia Nascimento, Leon Schurgers, Dina Simes

**Affiliations:** 1 Centro de Estudos e Desenvolvimento em Saúde, Campus de Gambelas, Universidade do Algarve, 8005-139 Faro, Portugal; epinto@ualg.pt (E.P.); tinascimento@ualg.pt (T.N.); 2 Algarve Biomedical Center Research Institute (ABC-RI), Campus de Gambelas, Universidade do Algarve, 8005-139 Faro, Portugal; 3 Centre of Marine Sciences (CCMAR), Campus de Gambelas, Universidade do Algarve, 8005-139 Faro, Portugal; caviegas@ualg.pt; 4 GenoGla Diagnostics, Centre of Marine Sciences (CCMAR), Campus de Gambelas, Universidade do Algarve, 8005-139 Faro, Portugal; 5 Research Centre for Tourism, Sustainability and Well-Being, CinTurs, Campus de Gambelas, Universidade do Algarve, 8005-139 Faro, Portugal; pventura@ualg.pt; 6 Department of Biochemistry, Cardiovascular Research Institute Maastricht, 6200 MD Maastricht, The Netherlands; l.schurgers@maastrichtuniversity.nl

**Keywords:** vitamin K, food frequency questionnaire, dietary intake, Mediterranean diet

## Abstract

Vitamin K is a multifunctional micronutrient essential for human health, and deficiency has been linked to multiple pathological conditions. In this study, we aimed to develop and validate a new food frequency questionnaire (FFQ) to estimate total vitamin K intake, over the course of a 30-day interval, in a Portuguese, Mediterranean-based, population. We conducted a prospective study in a non-random sample of 38 healthy adult volunteers. The FFQ was designed based on a validated Portuguese FFQ used in nationally representative studies and on literature reviews, to include foods containing ≥5 μg of vitamin K/100 g and foods with a lower vitamin K content, yet commonly included in a Mediterranean diet. Vitamin K intake was estimated from 24 h recalls and six days of food records. The final FFQ included 54 food items which, according to regression analyses, explains 90% of vitamin K intake. Mean differences in vitamin K intake based on food records (80 ± 47.7 μg/day) and on FFQ (96.5 ± 64.3 μg/day) were statistically non-significant. Further, we found a strong correlation between both methods (r = 0.7; *p* = 0.003). Our results suggest that our new FFQ is a valid instrument to assess the last 30 days of vitamin K intake in the Portuguese Mediterranean population.

## 1. Introduction

Vitamin K is a liposoluble vitamin essential for maintaining proper human health, and its deficiency has been linked to age-related diseases, such as cardiovascular disease, osteoarthritis, dementia, cognitive impairment, mobility disability, and frailty [1,2]. Vitamin K has been historically described as a co-factor for the gamma-glutamylcarboxylation of vitamin K-dependent proteins (VKDPs) with a multitude of functions in multiple body tissues and molecular processes. Vitamin K is considered an essential nutrient for blood coagulation and cardiovascular and bone health. In addition, vitamin K has been shown to exert novel roles independent of VKDPs carboxylation, such as anti-inflammatory, antioxidant, antiferroptosis, transcriptional regulator of osteoblastic genes, inhibition of tumor progression, and cognition promoter [1,2,3].

Naturally occurring vitamin K includes phylloquinone (vitamin K1) and several forms of menaquinones (MKn or vitamin K2). Vitamin K1 (VK1) is mostly found in photosynthetic organisms, including green leafy vegetables, herbs, algae, and vegetable oils. Vitamin K2 (VK2) is primarily produced by bacteria and is obtained through the consumption of meat, fermented foods, and dairy products [1,4]. Although, collectively, VK1 and VK2 comprise the generally described vitamin K, these isoforms differ not only in the source, but also in absorption rates, tissue distribution, bioavailability, and target activity [1]. Despite the fact that vitamin K can be recycled, that microbiota can produce VK2, and that MK-4 is a product of conversion from vitamin K at tissue level, vitamin K1 and K2 sources are mainly dietary [5,6]. According to the estimated dietary consumption, vitamin K1 accounts for 90% of the total vitamin K in the diet [7], but evidence from basic research and clinical studies has highlighted the importance of VK2. In fact, it was suggested that VK2 might account for 70% of total extrahepatic activity, while VK1 contributes only 5% [8]. Vitamin K has a recommended daily intake (RDI) based on the median intake of VK1 in American adults, and an adequate intake recommendation (adequate intake—AI) set at 120 μg/day for men and 90 μg/day for women [9]. In Europe, 75 μg vitamin K has been recommended as daily allowance (Commission Directive 2008/100/EC). Additionally, VK2 has been suggested to be suitable for consideration for a specific dietary recommendation intake [8].

While most diets are considered to contain an adequate amount of vitamin K, further research is needed, especially because the intake estimates of vitamin K are not consistent and mostly focused on VK1. Additionally, habitual intake is influenced by factors including food diversity, eating patterns, and different contents of vitamin K, which may vary with soil, climate, and agricultural conditions [10].

Food frequency questionnaires (FFQ) are commonly used to assess dietary intake in epidemiological studies because they are relatively easy to administer, are cost-effective, and are able to capture long-term habitual intake [11]. However, the validity of FFQ is dependent on the accuracy of the food composition database and the ability of participants to recall their intake over a specific period [12].

Validated FFQ to assess vitamin K intake exists, constructed using American populations, but these tools cannot be directly applied to a Mediterranean population, which traditionally follows an eating pattern where vitamin K-rich foods, such are leafy green vegetables and different types of cheeses, are frequently consumed.

The aim of this study was to develop and validate a new FFQ for assessing vitamin K intake, including VK1 and VK2, in a Mediterranean-based sample. To our knowledge, this is the first study to evaluate the validity of a FFQ specifically designed to assess vitamin K intake in this setting.

## 2. Materials and Methods

### 2.1. Study Design and Participants

We conducted a prospective study in a non-random sample of healthy adult volunteers, recruited by direct contact and through social media. Inclusion criteria were: (i) age ≥ 18 years and (ii) cognitive ability to fulfil the data collection tools. Exclusion criteria were: (i) currently under treatment for serious chronic illness, such as cancer or autoimmune diseases; (ii) diagnosis of a disease that impairs nutrition or food choice, such as Crohn’s disease, other malabsorption syndromes or food allergies; and (iii) on anticoagulant therapy.

Sample size calculation, according to methods proposed by Huley et al. [11], considering 90% power, 5% statistical significance, and a minimum predicted correlation coefficient of 0.55 between vitamin K intake estimated by FFQ and estimated by dietary recalls, yielded a minimum sample size of 30 individuals. Accounting for a 10% dropout rate, we set the minimum sample size at 35 individuals. On recruitment, 38 participants were eligible, and all proceeded to be a part of the study and concluded data collection.

All participants signed informed consent forms and this study was approved by the Ethics Committee of the University of Algarve, Portugal.

### 2.2. Data Collection

To apply the inclusion criteria, participants were recruited in a face-to-face interview and all details of the study were explained. Intake of vitamin K was assessed by one 24 h recall, conducted by a trained dietitian at the day of recruitment, and two self-fulfillment dietary records, comprising three, consecutive, complete days each. The first dietary record was completed in the days following recruitment, and the final record was completed one month later. One of the records was relative to weekend days (Friday, Saturday, and Sunday), and researchers contacted participants by e-mail to encourage them to fulfil the dietary records on the set dates.

Participants were asked to report the brand and type of each food item whenever possible. In the recruitment interview, participants were provided with forms to record their dietary intake, and any questions regarding this stage were addressed. Forms for the study were based on the EPIC-Norfolk dietary records forms [12] and in the Portuguese National Health and Physical Activity Survey [13] forms, which include detailed instructions and images showing portion sizes and common measurement cups and spoons. Researchers were available by phone at any time to address any additional questions during data collection.

After fulfilling the second dietary record, participants were also asked to complete the FFQ created for this study as detailed below. Participants were also asked to report their height, weight, education level, age, and gender.

### 2.3. Food Frequency Questionnaire (FFQ)

To construct the FFQ for this study, we based our food list on a validated Portuguese FFQ used in nationally representative studies [13]. Based on previous research [14,15,16] and on published food composition tables for vitamin K [7,17], we identified and selected foods with ≥5 μg of vitamin K/100 g of food and included them in the FFQ. Additionally, we identified foods that, despite having <5 μg of vitamin K/100 g of food, are commonly included in a Mediterranean diet, such as some types of soft cheese, boiled chickpeas, pumpkin, bell pepper, mackerel, sardines, almonds, walnuts, and coffee [18,19]. The initial FFQ included a total of 103 food items. Statistical analysis for this validation study, detailed in the results, allowed us to propose a final FFQ with 54 food items.

Participants were asked to report the frequency of intake in the last month of all listed food items by choosing one of eight frequency options: never or less than once per month, 1–3 per month, 1 per week, 2–4 per week, 5–6 per week, 1 per day, 2–3 per day, 4+ per day.

All food items were presented with a photograph with a recommended portion weight, also used in the Portuguese National Health and Physical Activity Survey [13], and participants reported if their average portion was the same, smaller (defined as 0.5 times smaller), or larger (1.5 times larger) than the recommended size.

Vitamin K intake for each food item was computed based on the following formula: nutrient content in portion × average portion size x frequency conversion factor. The conversion factors were those proposed in previous research on this subject [20,21,22] and are presented in Table 1.

Vitamin K intake was calculated using a food composition database constructed for this study in Microsoft Excel, which included data on vitamin K content of foods from previous research [7] and data from both McCance and Widdowson’s and the United States Department of Agriculture’s food composition databases [17,23].

### 2.4. Statistical Analysis

Data were analyzed using IBM SPSS for Windows (version 29.0, 2022, IBM Corporation). Mean, median, standard deviation (SD), and interquartile range (IQR) were computed whenever appropriate, and we used the Shapiro–Wilk test to assess adherence to the normal distribution of variables regarding vitamin K intake. As the distribution of these variables was non-Gaussian for both food records and FFQ, data were log-transformed for some statistical inference tests.

Agreement was analyzed using Student’s *t*-tests and plotted according to the Bland–Altman method [24]. We also computed Pearson’s correlation coefficient on the log transformed data.

Statistical significance for all procedures was set at 0.05.

## 3. Results

### 3.1. Participants

All participants (*n* = 38) followed the data collection procedures for the study and completed the food records and the FFQ.

Participants were predominantly females (*n* = 23; 60.5%). All of the men (*n* = 15; 100%) and 48% of the women (*n* = 11) had a college level degree. Mean body mass index (BMI) was 24.6 ± 0.6 kg/m^2^ and ages ranged between 18 and 65 years old. Female participants were significantly older (*p* = 0.004) and with a lower educational level than males (*p* = 0.002). Table 2 summarizes the characteristics of participants.

### 3.2. Food Frequency Questionnaire Items

To analyze the adequacy of the food item list in the FFQ, we conducted multiple regression analyses, with a stepwise method, using vitamin K intake as the dependent variable and all other food items as independent variables. The food items that contributed cumulatively up to 90% of vitamin K intake in a statistically significant model (F = 7.5, *p* = 0.004) were retained for the final version of the FFQ. From the initial list of 103 food items, we eliminated 43, and thus included 54 food items in our FFQ. The included food items are shown in Table 3.

### 3.3. Comparison between Food Records and Food Frequency Questionnaire

Mean vitamin K intake was 80 μg (±47.7) according to food records and 96.5 μg (±64.3) according to the FFQ. Vitamin K estimates from the FFQ were, on average, 16.5 μg (±82.57) higher than estimates from food records. This difference is statistically non-significant (*p* = 0.226), supporting the existence of proper absolute agreement between both methods. The same results are observed in a paired Student’s *t*-test on log-transformed vitamin K intake data (*p* = 0.293). We also found a strong and statistically significant correlation (r = 0.697; *p* = 0.003) between the FFQ and diet records, which suggests a good relative agreement and that the FFQ is a valid instrument to assess vitamin K intake in this population. Table 4 summarizes the results from the comparison between FFQ and food records.

**Figure 1:**
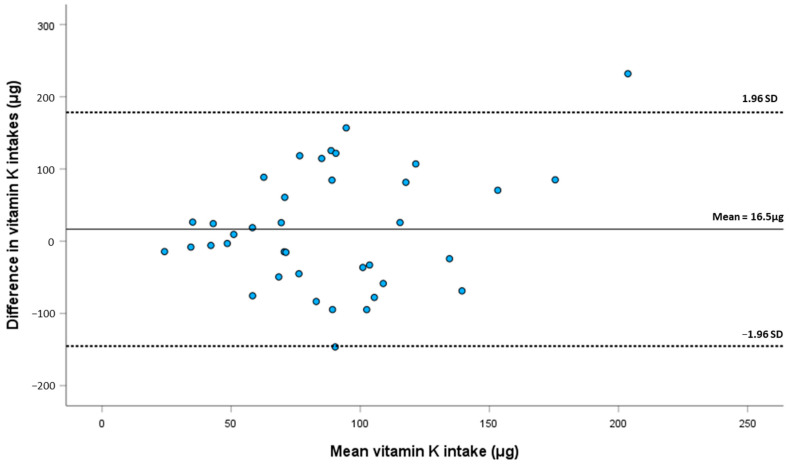
Bland–Altman plot to assess agreement in vitamin K intake between food records and the food frequency questionnaire.

We constructed a Bland–Altman plot to assess agreement by computing mean vitamin K intake from FFQ data and six days of food records, and plotting this with the difference in mean intake obtained with these two methods. The plot shows that most participants fall in the acceptable limits of agreement in individual differences between both dietary estimate methods (Figure 1).

Following the methods proposed by Doğan [25] and Ludbrook [26], we conducted a simple linear regression analysis using the Bland–Altman plot data, considering the difference in mean vitamin K intake as the dependent variable and mean vitamin K intake as independent variable. This analysis showed a regression where mean differences are not significantly different than zero (F = 0.004; *p* = 0.959) and a statistically non-significant (*p* = 0.949) β_1_ coefficient, very close to the null value (β_1_ = −0.024), suggesting that there is no proportional bias. This shows that the difference in values resulting from the two methods does not increase or decrease in proportion to the average values.

## 4. Discussion

The literature suggests that FFQ are valid, reliable, and easy-to-use tools in populational studies, but their accuracy in estimating some nutrients, particularly micronutrients, is still the subject of ongoing research [22,27,28,29]. Thus, the development of a valid FFQ for vitamin K that can be used in surveys, cohort studies, and other populational assessments can constitute worthwhile research, especially if the FFQ is aimed at populations that are associated with a specific dietary pattern, which includes vitamin K-rich foods.

Our study allowed the development of a valid FFQ for the assessment of vitamin K intake in a sample of Portuguese adults, with a very low cost of administration and processing, and with low respondent burden. Moreover, this FFQ is directed to estimate VK1 and VK2 intake as a more representative measurement of vitamin K status. Most FFQs developed to estimate the dietary consumption of vitamin K have specifically focused on VK1 mainly because, in the Western diet, it accounts for nearly 90% of total vitamin K intake [7]. However, accumulating evidence from basic research and clinical studies has highlighted the health beneficial effects of VK2, particularly due to its long half-life and extrahepatic distribution. While VK1 is preferentially accumulated in the liver and is poorly retained in the organism with a half-life time of 1–2 h, VK2 with a half-life time of 68 h is available to extrahepatic tissues through circulation, resulting in an increased bioavailability of the whole body [1,2]. Additionally, in terms of functionality, VK2 (particularly MK-7 and MK-4) has been shown to have a higher bioactivity than VK1 in different molecular processes, such as gamma-glutamylcarboxylation cofactor, inhibitory effect on bone resorption, antioxidant, activator of sphingolipid metabolism, and anticancer [30,31,32,33,34,35]. In fact, it was suggested that VK2 is the major active form of vitamin K, accounting for 70% of total extrahepatic activity, while VK1 contributes only 5%, and that the beneficial effects of VK2 are not covered by current RDI guidelines [8]. However, it should be noted that MK-4 is a result of vitamin K conversion and is dependent from VK1 intake, and that MK-4 is present in most extrahepatic tissues [5,6,36,37]. In this context, more attention should be given to the dietary intake of both VK1 and VK2.

Although we recorded some overestimation (16.5 ± 82.57 μg) of vitamin K intake when compared with food records, this overestimation was statistically non-significant and on par with the variability which, according to previous research, is to be expected [38,39,40,41]. Our FFQ showed an adequate relative agreement with food records, with a correlation coefficient of 0.697, which is above the minimum threshold of 0.4 proposed for FFQ validation studies [22,42]. Previous FFQ studies regarding vitamin K intake report correlations from 0.5 to 0.8 [15,16,40,43,44,45]. The Bland–Altman method of analysis also indicates good relative agreement, with one outlier participant above the 95% agreement limit, and one participant in the borderline of the lower agreement limit. Although this study is not intended to evaluate vitamin K intake in our population, the mean vitamin K of 96.5 ± 64.3 μg/day in our sample is within the RDI established for the American population and above the 75 μg/day recommended by the European Commission.

We identified several limitations of this study and propose future research directions in this topic. Although VK1 intake has been suggested to not vary significantly with season [46], we cannot rule out the interference of the seasonal nature of dietary patterns, particularly when considering a Mediterranean-based diet. We included items in the FFQ food list that cover the wide variety of products that are common in all harvesting seasons, but data were collected in springtime. This can imply that the final food list that derived from the regression analysis may not include items that provide an important intake of vitamin K, because these are mainly consumed during winter. We believe that wintertime products are represented in the final list, but additional studies to confirm this, conducted during colder months, are needed. Some research suggests that there is a high within-person variation in the intake of vitamin K [41] and green vegetables [42,43]. Therefore, additional studies assessing the reproducibility of our FFQ should also be undertaken, as we did not assess this parameter. Although the sample size of our study is adequate to compare methods of assessing dietary intake, our participants constituted a non-probabilistic, convenience sample, mainly composed of adults with a high educational level. Ages ranged from 18 to 65 years of age and, therefore, our data do not allow us to assess the validity of our FFQ in older adults, or in individuals with different sociodemographic characteristics, thereby compromising the generalization of our results.

It is also important to note that we focused on collecting vitamin K intake during a period of one month. The initial 24 h recall and the food records that were used to establish usual intake were collected during a 30-day interval and the FFQ was specifically constructed in such a way that participants recall their intake for an equivalent period. This limited timespan can favor recall, as participants’ memory of recent food intake can contribute to the good agreement shown in the data. The aim of the FFQ we developed was to assess short-term intake and, thus, we cannot infer or extrapolate to longer periods of intake recall and propose this to be taken into account in future studies with this tool.

Overall, we constructed a valid, practical, and cost-effective tool to estimate vitamin K intake for a 30-day period. Our FFQ showed good absolute and relative agreement with food records and includes a list of food products that account for most of the VK1 and VK2 intake in a Mediterranean dietary pattern in the Portuguese population. Additional research is needed to assess the reproducibility of this tool to confirm that seasonality does not affect its validity, and to test its applicability to different populations.

## Figures and Tables

**Table 1:** Frequency intake and conversion factors.

Intake Frequency	Conversion Factor
Never or less than once per month	0.02
1–3 per month	0.07
1 per week	0.14
2–4 per week	0.43
5–6 per week	0.79
1 per day	1
2–3 per day	2.5
4+ per day	4

**Table 2:** Characteristics for all participants by gender.

Characteristics		All		Males		Females		*p*-Value
		(*n* = 38)		(*n* = 15)		(*n* = 23)		
**Age (years)**	**Mean (±SD)**	**37**	**(±12.2)**	**31**	(±4.5)	41	(±14)	**0.004** ^a^
Median (IQR)		36.5	(19)	31	(8)	44	(27)	
BMI (kg/m^2^)	Mean (±SD)	24.6	(±0.6)	24.8	(±0.3)	24.5	(±0.7)	
Median (IQR)		24.6	(0.5)	24.7	(0.6)	24.6	(0.6)	0.051 ^b^
Education (level):								
Primary school (4 years); *n* (%)		2	(5%)	-		2	(9%)	**0.002** ^c^
Middle school (5–9 years); *n* (%)		2	(5%)	-		2	(9%)	
High school (10–12 years); *n* (%)		8	(21%)	-		8	(35%)	
College level degree; *n* (%)		26	(69%)	15	(100%)	11	(48%)	

BMI—Body mass index; SD—standard deviation; IQR—interquartile range. Gender differences computed with: ^a^—Student’s *t*-test; ^b^—Mann–Whitney’s test; ^c^—Fisher–Freeman–Halton exact test; Statistical significance (*p* < 0.05) is **boldfaced**.

**Table 3:** Food items in the final food frequency questionnaire.

Category	Food Item
Meat, fish, and eggs	Tuna in oil, drained; Beef, minced meat or hamburger; Cold cuts (ham, mortadella, chorizo, etc.); Mackerel, horse mackerel or small fish; Scrambled or fried egg
Grains and tubers	Homemade or restaurant French fries; Packaged potato chips or corn snacks; Homemade bread
Desserts and sweets	Cookies, biscuits; Cake, tart or other pastry product (croissant, etc.); Milk chocolate
Fruits	Plum, cherry; Almond, walnut, hazelnut; Peanut, cashew; Dried figs; Apple, pear; Strawberry; Nectarines; Peach; Grapes; Avocado
Vegetables and pulses	Pumpkin; Watercress, arugula; Lettuce or mixed green salad; Leek; Eggplant; Broccoli; Carrot; Coriander or parsley; Kale; Cabbage, collard greens, brussels sprouts; Cauliflower; Zucchini; Peas; Asparagus, green beans; Spinach; Cooked beans; Cooked chickpeas; Greens, creamed spinach, green vegetable puree; Cucumber; Green pepper; Red pepper; Tomato
Fats and oils	Olive oil; Butter, margarine or vegetable spread, mayonnaise; Cooking oil
Other	Espresso coffee
Meals	Lasagna or pasta; Stir-fry or stew with vegetables and/or pasta
Dairy products	Creamy cheese (Brie, Camembert) or Serra da Estrela cheese; Cream cheese, spreadable; Cured cow or sheep cheese, Edam cheese; Goat cheese; Cottage or fresh cheese

**Table 4:** Vitamin K intake according to food records and food frequency questionnaire.

		Food Records		FFQ		*p*-Value
Vitamin K intake (μg)	Mean (±SD)	80	(±47.7)	96.5	(±64.3)	0.293 ^a,b^
Median (IQR)		77.4	(84.5)	80.9	(89.4)	
Minimum		16.1		17		
Maximum		173.8		319		
Difference between FFQ and food records: Mean (±SD); (95% CI)		16.5 (±82.57); (−10.7, 43.6)				0.226 ^b^
Correlation between FFQ and food:		0.697				0.003 ^a^

SD—Standard deviation; IQR—interquartile range; CI—confidence interval; FFQ—food frequency questionnaire. ^a^—*p*-values corresponding to analysis on log-transformed variables; ^b^—Student’s *t*-test.

## Data Availability

Data available on request due to privacy restrictions.
